# The Role of Value Stream Mapping in Healthcare Services: A Scoping Review

**DOI:** 10.3390/ijerph18030951

**Published:** 2021-01-22

**Authors:** Juan A. Marin-Garcia, Pilar I. Vidal-Carreras, Julio J. Garcia-Sabater

**Affiliations:** ROGLE, Department of Organización de Empresas, Universitat Politècnica de València, Valencia s/n, 46021 Valencia, Spain; pivicar@omp.upv.es (P.I.V.-C.); jugarsa@omp.upv.es (J.J.G.-S.)

**Keywords:** lean healthcare, Value Stream Mapping (VSM), patient flow, process improvement, systematic literature review, sustainability, scoping review, bibliometric, bibliometrix, primary care, secondary care

## Abstract

Lean healthcare aims to manage and improve the processes in the healthcare sector by eliminating everything that adds no value by improving quality of services, ensuring patient safety and facilitating health professionals’ work to achieve a flexible and reliable organization. Value Stream Mapping (VSM) is considered the starting point of any lean implementation. Some papers report applications of VSM in healthcare services, but there has been less attention paid to their contribution on sustainability indicators. The purpose of this work is to analyze the role of VSM in this context. To do so, a scoping review of works from recent years (2015 to 2019) was done. The results show that most applications of VSM reported are in the tertiary level of care, and the United States of America (USA) is the country which leads most of the applications published. In relation with the development of VSM, a heterogeneity in the maps and the sustainability indicators is remarkable. Moreover, only operational and social sustainability indicators are commonly included. We can conclude that more standardization is required in the development of the VSM in the healthcare sector, also including the environmental indicators.

## 1. Introduction

The lean manufacturing concept has been applied for years now in the healthcare services sector (primary care centers or specialist centers and hospitals). Lean healthcare can be considered a management philosophy for developing a hospital culture characterized by increasing the satisfaction of patients and other stakeholders through continuous improvements, in which all employees actively participate in identifying and reducing non value-adding activities (waste) [1,2].

Different techniques can be used to help the continuous improvement process and to deploy lean manufacturing [3,4,5,6]. One of these is Value Stream Mapping (VSM), which has been traditionally considered a basis or a basic pillar to deploy others [7,8,9]. When the healthcare institutions apply VSM, their aim is to develop a culture that promotes the satisfaction of patients, healthcare staff and healthcare managers by means of continuous improvement, characterized by the personnel’s engagement (managers, doctors, female nurses, lab technicians, hospital porters, office staff and outsourced workers) to detect areas than can improve [8]. 

Until 2015, very few works had investigated using VSM in different sectors and even fewer specifically in healthcare services [10]. We are unaware of whether the situation has changed in recent years. We will also try to find out how to adapt the VSM methodology to design future states, as is proposed by Rother [11], in healthcare services. The construction of the map of the future state is based on the design of material management tools (for example Kanban), which must be adapted in some way for patient management. In addition, papers are beginning to appear that address the problem of environmental sustainability in the design of the map [12]. Our research will shed some light on these matters.

Integrating published knowledge is a vital contribution to the development of the scientific field [13]. It allows researchers to know, on the one hand, the real evidence currently available and, on the other hand, which limitations impact the available published research and the possible research niches that future research can deal with. Indeed, in relation to VSM, our research will provide researchers with a list of recent fieldwork done on this theme and we help to verify if the demand of studies on this matter has been covered according to previous literature reviews [8,14].

The remainder of this paper is organized as follows. Section 2 presents the theorical framework for this work, defining the VSM concepts and healthcare services sector to be used herein. Conclusions reached by previous systematic reviews about the topic are exposed also in this section. Section 2 finishes enumerating the research questions to solve with the work. Section 3 describes the materials and methods for the systematic literature review. Section 4 presents the results obtained, solving the former research questions, and the discussion is presented in Section 5. Finally, in the last section, the conclusions are drawn, as well as the limitations and the future work.

## 2. Theoretical Framework 

Value Stream Mapping is a diagnostic technique of the process’ present status. Although different VSM versions exist [10,15,16], we center on what Rother and Shook propose [11], which is probably the most widespread version in industrial settings linked with continuous improvement, although we lack evidence that this is the case in other contexts.

The main peculiarity of Rother and Shook’s version [11] lies in it graphically showing in the same diagram the information flow and materials flow [8,10,17] needed to complete a project, a product or a service [17,18]. This version normally focuses on an organization’s internal operations by showing the main steps from raw material warehouses to the deliveries point or the supply point to clients [17,18]. Nonetheless, some adapted versions specifically represent all the supply chain elements and intersections, in which several plants and/or organizations intervene. This VSM version is characterized by using standardized symbols [8,17] to present the flow of a family of products or services now and in the future. Other VSM versions are available [19,20,21], but essentially share the same elements. The way in which the described VSM is run can allow added-value and non-added-value activities to not only be identified, but improvement actions to start [8,10,18].

With our research, we wish to deal with any healthcare level (primary care, specialist medical consultations, hospitals, reference centers for uncommon diseases and geriatric or disability care care). We explore using VSM in organizations in any country in the world regardless of them being public, private, or not-for-profit foundations. We focus on healthcare services with patients and include neither the pharmaceutical industry nor government/non-government public health structures (e.g., ministries, the Red Cross or the like). Primary care focuses on general practitioners and outpatient services (nursing and pharmacy). Specialties like internal medicine, neurology, general surgery, cardiology, immunology, oncology, etc., make up a different healthcare level (secondary care), which, according to the health system, may have reference centers. This secondary care is normally provided in specific clinics but can also take place in hospitals (tertiary care). In some public health systems, specialists can only be accessed by a primary care doctor or through emergency services [22,23,24].

One of the main goals pursued with the VSM is to improve some key performance indicators (KPI) of the organization. In this work, we are going to group them into three main pillars: operational, environmental and social indicators [25,26,27,28,29]. For example, for operational indicators, among others, the following have been used [24,29,30,31,32]: clinical quality, cost reduction, patient safety, organizational responsiveness, lead time, delivery performance, process time, inventory level, equipment performance, reducing delays, error rate, service quality and patient satisfaction, and for environmental indicators [26,30]: waste reduction, energy consumption, resource reduction, carbon footprint, carbon value efficiency and fossil fuel reserve emissions. Finally, some examples for social indicators are [30,33,34,35]: employee well-being, work–life balance, employee satisfaction, work accidents, turnover rate, etc.

The published literature reviews about VSM that we found center on analyzing several economic activities [10,36,37,38] in which manufacturing industries predominate [27,39]. Many of these reviews [40,41] do not deal with specific works on VSM, but about lean manufacturing in general, although others cover the healthcare sector [42,43]. We also found that some reviews center on the pharmaceutical sector [44]. Former research seems to indicate that VSM allows the transparency of the process to improve and to make it more comprehensible to the stakeholders involved in it [8,10] by reducing not only the process lead time [10], but also inventories [10]. However, these results mostly derive from repetitive production contexts (related to the automobile or the consumer electronics sector, or their auxiliary industries), and normally from Anglo-Saxon countries [31]. It would appear that there are not enough available publications to generalize these results to all kinds of contexts. Some published studies indicate that the barriers which prevent such tools being used can outbalance facilitators in public services contexts [45].

As previously mentioned, a series of former papers roughly cover VSM in research studies that concentrate on lean. Some consider that VSM is an interesting future research line [46]. Others [47,48] cite it as a lean tool, but do not go into it in much detail. Only four studies specifically focus on VSM in the healthcare services sector. Bucci et al. [49] conclude that emergency departments that employ VSM successfully remove non-value-added activities through managing patient flows. Moraros, Lemstra and Nwankwo [50] apply a search strategy centered on lean or Kaizen with health and focus on articles published from 2007 to 2014. In the 22 selected articles, the main interventions that they mention are: Waste analysis (*n* = 22), Plan Do Check Act (PDCA)/Define Mesure Analyse Improve Control (DMAIC)/A3 (*n* = 15), Value Stream Map (VSM) (*n* = 10), standard operations procedure or training (*n* = 8), work redesign (*n* = 4) and triage (*n* = 3). A clear association comes out between waste analysis and PDCA/DMAIC, and with VSM, as these interventions tend to overlap in the studied works. The main indicators analyzed as outcomes are operational (length of stay/wait time and productivity/efficiency, followed by work engagement, trust and movement reduction). Nowak et al. [14] also report having searched many databases, but they adopt a much more specific strategy to seek VSM. These authors’ studies comprise articles published between 2000 and 2015, which include the pre- and post-results when VSM is used as an intervention, and they implement future VSM solutions. These authors also include 22 articles (which differ from those selected by Moraros, Lemstra and Nwankwo [50]). The interventions related to VSM are training (*n* = 1), relocation of workers (*n* = 1), layout changes (*n* = 1), triage (*n* = 8) and waste analysis (*n* = 16). Outcome indicators are length of stay (*n* = 15), staff satisfaction (*n* = 2) and pay satisfaction (*n* = 2). Without being able to draw evidence-based conclusions, the authors indicate that VSM seems to have positive effects on service quality and reducing non-value-added activities, and they clearly observe shorter waiting times and, consequently, length of stay. However, these improvements might be due to both VSM and some of its related interventions, and it is not possible to specify any effects. Vidal-Carreras et al. [8] focus on searching in the Web of Science (WOS) databases. They specify a narrow VSM-focused strategy and healthcare for works published up to 2014 and include 18 articles. An overwhelming majority are from Anglo-Saxon countries. These authors also indicate the number of publications with positive results, which seems disproportionate compared to the published failure cases (in both the first implementation and the long cycle).

Of the four recent above-mentioned reviews, one [49] centers only on emergency departments and does not cover any other healthcare services sector. The other three reviews [8,14,50] coincide in stressing that they are unable to draw conclusions about the effects that VSM interventions have on the healthcare services sector for several reasons: very few published works, and both interventions and outcomes are too heterogeneous insofar as they cannot aggregate outcomes and the VSM intervention is rarely presented separately. So, the observed effects might be due to any of the VSM-related interventions. Therefore, all three conclude that more field research about VSM in the healthcare service sector is necessary. Moreover, none of the reported articles in these four reviews have analyzed the key performance indicators (KPI) related to environmental or social categories. Finally, all these reviews have analyzed information published until 2015 as the most recent articles.

As a few years have elapsed since the date of the latest references included in the reviews we found [8,14], we believe it is worth performing a new systematic review about VSM in healthcare services and checking if new works have since been published that contribute new evidence and overcome some of the limitations of the previous systematic reviews in this study area.

Our systematic review intends to answer some questions inspired in the field research conducted by the authors of the present work and of previously published research works [23,25,51,52]:

1. How much research on VSM in healthcare has been published in the period 2015–2019?
2. In which health areas has the VSM methodology been applied in the published articles?
3. What sustainability indicators are considered when applying the VSM methodology?
4. Is VSM employed alone or along with other lean tools? If so, which ones?
5. What is the common VSM version used in healthcare sector publications?

Moreover, we can reflect on the existing research gap for VSM when it is applied to the current healthcare services sector, and which research questions about VSM remain open.

## 3. Materials and Methods 

Our work is framed within descriptive literature review and scoping review typologies [35,53]. The procedure followed for our systematic literature review is based on previous recommendations and working practices according to other authors [25,45,54,55,56,57,58,59,60]. The methodology can be consulted in detail in the published research protocol [61].

▪ Inclusion criteria
  1. Research works published between 2015 and 2019 in the journals or conferences indexed in Web of Science (WOS) databases
  2. Research works that investigate applying VSM to public or private clinics or hospitals anywhere in the world.
  3. Research works written in English, Spanish, French, German, Italian or Portuguese.
▪ Exclusion criteria
  4. Book chapters, dissertations and other sources that are not easy to access and with no guaranteed review process (e.g., consultant firms’ reports).
  5. Applications in health areas that are not the object of our research, such as the pharmaceutical industry or official organizations or institutions (Red Cross, Ministry of Health, World Health Organization (WHO), Médecins Sans Frontières, etc.). For instance:
    - Ha, C., Taylor, C., & Modi, J. R. (2016). Mass Vaccinations at the United States Naval Academy. HEALTH SECURITY, 14(6), 382–388. https://doi.org/10.1089/hs.2016.0030
  6. The research work makes no clear contribution with VSM, but only appears indirectly as merely another lean manufacturing technique, or as a continuous improvement or processes improvement technique. For instance:
    - Fung-Kee-Fung, M., Maziak, D. E., Pantarotto, J. R., Smylie, J., Taylor, L., Timlin, T., … Stewart, D. J. (2018). Regional process redesign of lung cancer care: a learning health system pilot project. CURRENT ONCOLOGY, 25(1), 59–66. https://doi.org/10.3747/co.25.3719
    - Furterer, S. L. (2018). Applying Lean Six Sigma methods to reduce length of stay in a hospital’s emergency department. QUALITY ENGINEERING, 30(3), 389–404. https://doi.org/10.1080/08982112.2018.1464657
    - Al Owad, A., Samaranayake, P., Karim, A., & Ahsan, K. B. (2018). An integrated lean methodology for improving patient flow in an emergency department—case study of a Saudi Arabian hospital. PRODUCTION PLANNING & CONTROL, 29(13), 1058–1081. https://doi.org/10.1080/09537287.2018.1511870
    - Basta, Y. L., Zwetsloot, I. M., Klinkenbijl, J. H. G., Rohof, T., Monster, M. M. C., Fockens, P., & Tytgat, K. M. A. J. (2016). Decreasing the dispatch time of medical reports sent from hospital to primary care with Lean Six Sigma. JOURNAL OF EVALUATION IN CLINICAL PRACTICE, 22(5), 690–698. https://doi.org/10.1111/jep.12518
  7. Articles containing only one page or two, with barely any information to be subsequently analyzed. For instance:
    - Rodrigues, D. M., Sadeghi, M., Bernstein, M., & Yong, E. (2017). Utilization of quality improvement strategies in the inpatient endoscopy setting at a tertiary care educational hospital. GASTROENTEROLOGY, 152(5, 1), S221–S222. https://doi.org/10.1016/S0016-5085(17)31037-5
▪ Search strategy

To construct an automatic search strategy, we connected two chains. The first one adopted works related to the healthcare services sector. For this purpose, we started with the strategy adopted by previous works [8,24] and we improved it in several ways. On the one hand, we simplified it by removing redundancies and using proximity (NEAR/WITHIN) or truncation (*) operators. On the other hand, we included synonyms taken from other reviews [14,62,63] to avoid false positives. 

The second chain included works related to VSM. To this end, we considered an improved strategy compared to that employed in former works works [8,14,63]. In it, we removed redundancies and put the potential of proximity (NEAR/WITHIN) or truncation (*) operators to good use. This allowed us to obtain simpler and more comprehensible structures (which can help us to avoid the logical errors made in connecting operators). We also included new synonyms and terms that could provide us with research works found in those contexts in which VSM is likely to have been used. We did not include the term “VSM” because we checked [62] that it introduced many false-positives and did not help to avoid false-negatives. WOS search from 11 February 2020 (TS = (((“value stream” NEAR/2 (Map* OR analysis OR Design OR management OR lean OR Chain OR “six sigma”)) OR ((Lean OR “Value Stream”) AND ((“define-measure-analyze-improve-control” OR dmaic) OR (flow* NEAR/2 (patient OR information OR process OR material OR work OR production OR waste))))) AND (((health* OR care OR medical OR nursing) NEAR/3 (system* OR institution* OR organisation* OR organization* OR facilit OR Social OR setting* OR enterprise OR service* OR social OR centers OR department*)) OR (Physician* or doctor) NEAR/3 (clinic or Office) OR surgery OR “clinical operation” OR “operating rooms” OR “SURGICAL clinics” OR “emergency room” OR pharmacy OR hospital OR hospitals OR clinic* OR “tertiary care” OR “primary care” OR “secondary care” OR “emergency department” OR (home NEAR/2 (retirement OR nursing OR “old people’s” OR “senior-citizens” OR disabled OR handicap*)))). Databases = WOS, CCC, DIIDW, KJD, MEDLINE, RSCI and SCIELO, with timespan = 2015–2019 and search language = Auto, leading to 244 results.

We compared the results we obtained with our strategy to those adopted by Vidal-Carreras et al. [8], such as scoping study [64], and to all their works obtained by our selection process.

**Figure 1:**
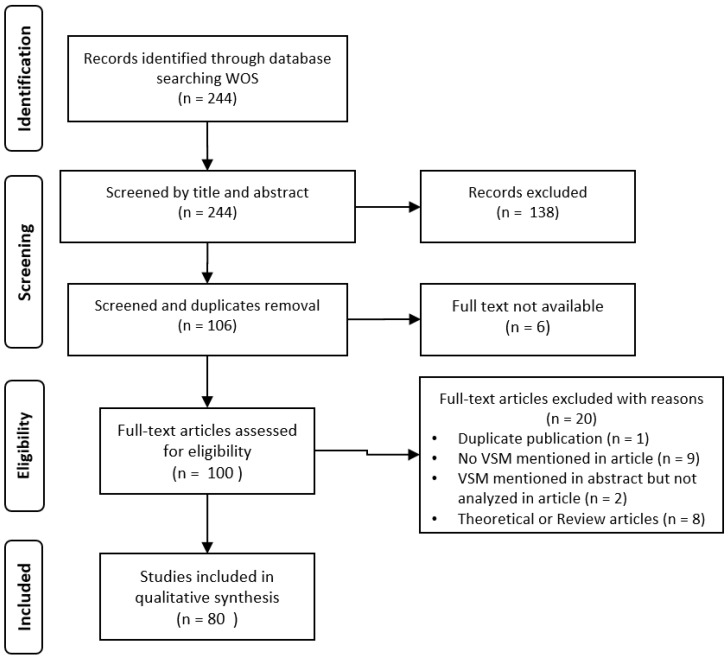
PRISMA diagram.

To manually filter the results (by analyzing the information found in the title and abstract), the last two authors worked independently on the WOS result. The first author overcame any divergences that had not been solved by reaching a consensus with the other two authors [60,62,65,66]. Figure 1 shows the Preferred Reporting Items for Systematic Reviews and Meta-Analyses (PRISMA) diagram with the selection process summary.

By reading the full text of the eligible articles, the three authors collected information for the codes representing the research questions by filling in a card for all the references [67,68,69,70,71,72]. The three authors coded five articles to verify the agreement of collecting information and to reach a consensus about codes. The other articles were independently coded (25–30 articles per author) and this information was acquired:

- Citation: specifying the author, year and titles of the article (e.g., “Dogan, N. O., & Unutulmaz, O. (2016). Lean production in healthcare: simulation-based value stream mapping in the physical therapy and rehabilitation department of a public hospital. Total Quality Management and Business Excellence, 27(1–2), 64–80. https://doi.org/10.1080/14783363.2014.945312”).
- Objectives: A brief description of the article’s objectives.
- Research type: literature review, survey/interview (cross-sectional), longitudinal (intervention or observation).
- Population: country or geographic region of which the data sample is representative.
- Sample (for surveys or multiple cases): if they are longitudinal, the hospital’s name.
- Year: indicating the year when the data or data sources employed in the research were collected.
- Questions: identify the main ideas set out in the articles.
- Healthcare sector: primary care consultation, consultation with a specialist, hospital, geriatric, others.
- Unit: emergency services, cardiology, traumatology, etc.
- Current state VSM (Yes/No).
- Future state VSM (Yes/No).
- Suggest improvement actions.
- Other TQM or lean interventions used with VSM.
- Indicators Baseline Value (Yes/No), Post Value (Yes/No).
- Indicators: operational/social–human resources/environmental.
- Limitations that the article indicates.

If a doubt arose, it was solved by the three authors reaching a consensus. The R Bibliometrix package was used for the bibliometrics analysis [52,56,73].

## 4. Results 

This section deals with solving the research questions.

### 4.1. RQ1: How Much VSM Research in the Healthcare Sector Has Been Published? 

As previously mentioned, 80 documents were finally selected to be included in this work, and the bibliometrics analysis was performed on them. Table 1 summarizes our sample, and acts as an overview of the research conducted about VSM in the health sector in 2015–2019. The typology of these 80 documents included: 71 papers published in journals, 8 proceeding papers and 1 editorial article. We found 67 different sources of the works we selected. Our sample contains 218 different keywords. The average number of citations per document is 4.8.

The analysis performed by the authors shows that the sample contained 408 different authors, although some authors had published more than one work. Of the 80 documents, we highlight that only two were written by a single author. The mean number of co-authors per document is 5.1.

After offering this overview of the research conducted on VSM in the health sector since 2015, we performed a detailed analysis of the number of articles per year, the number of articles per journal, the number of articles per author, the number of articles per country and the most cited journals and works.

**Figure 2:**
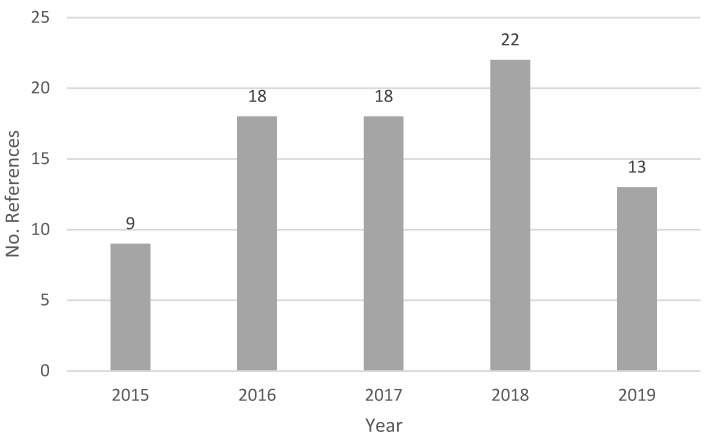
Number of references per year from 2015 to 2019.

The time distribution of publications is found in Figure 2. 

As we can see (Figure 2), the trend in 2015 and 2018 has grown, but it remained stable in 2016 and 2017. However, a considerable reduction was noted in 2019. Part of this reduction might be due to delays in journals’ indexing because, according to the citations found, this topic does not seem to have matured. Some authors did not doubt classifying VSM in 2017 as highly relevant [74,75].

**Figure 3:**
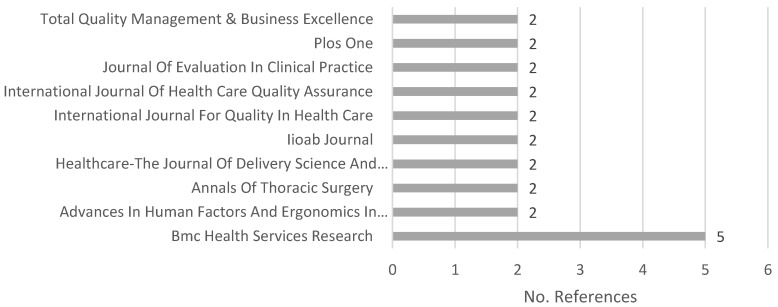
Number of references published per source.

Figure 3 depicts the number of publications in each source and/or journal. 

As shown in Table 1, 67 different sources appeared in our sample. Figure 3 shows the 10 journals with the most published articles. The journal BMC Health Services Research stands out in the set of sources with five articles about the subject, followed by nine journals with two publications each on this subject. The 57 remaining sources in the sample only presented one publication on the subject of our review for the 2015–2019 period.

As previously mentioned, 408 different authors appeared in the summary of our sample, along with a mean of 0.196 articles/per author. Table 2 shows the 13 authors with more than one published article. The remaining 395 authors only appear in one article. 

**Figure 4:**
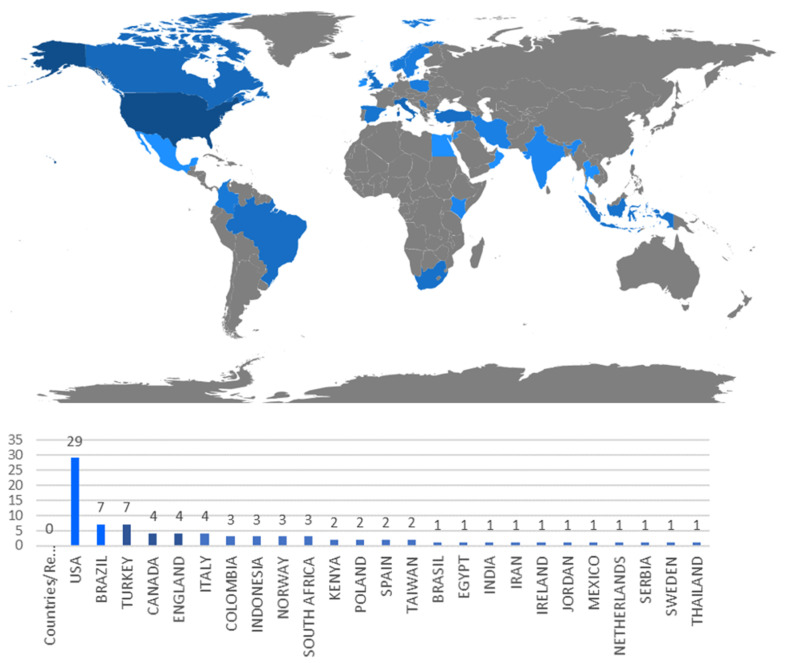
References per country of the main author.

Figure 4 presents the number of references per country of the main author. According to Figure 4, the USA is the leading country as it is the country in which more references are published according the affiliation data of the main author. It is followed at a considerable distance by Brazil and Turkey with seven works, and then by the UK, Canada and Italy with four. All the other works are distributed among the other countries.

Table 3 shows the most cited works of our sample. The two most cited works are those by Hicks, McGovern, Prior and Smith [76] from 2015, with 43 citations and a 7.2 average number of citations/year, and that by Henrique, Rentes, Godinho Filho and Esposto [77] from 2016, with 38 citations and a 7.6 average number of citations/year. They are followed by a work by Improta et al. [78] from 2015, with 22 citations and a 3.7 average number of citations/year. 

**Figure 5:**
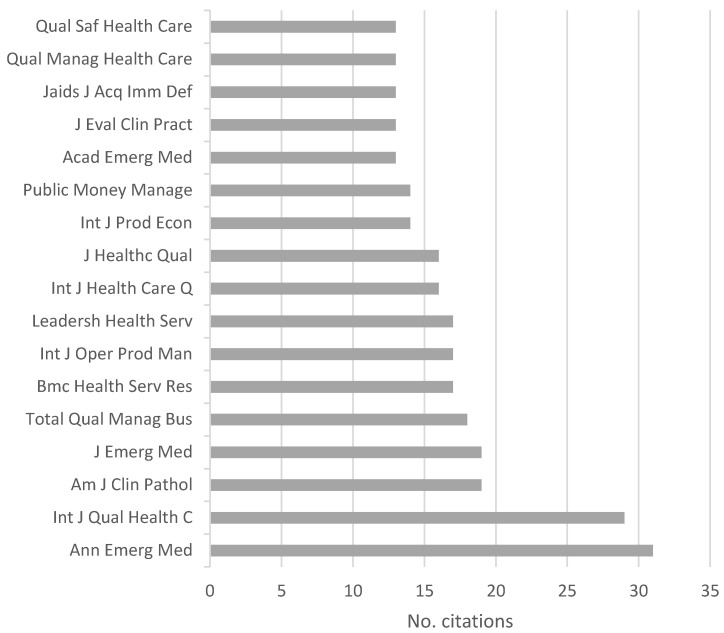
Number of citations per source.

Figure 5 summarizes the most cited journals, with more than 13 citations among the 80 articles. Seventeen journals form the most cited ones, of which we highlight the journals Ann Emerg Med with 31 citations and Int J Qual Health C with 29. The journal BMC Health Services Research that leads the analysis of quantity of papers included in our review (Figure 3), in this case, has 17 citations and occupies the sixth position.

**Figure 6:**
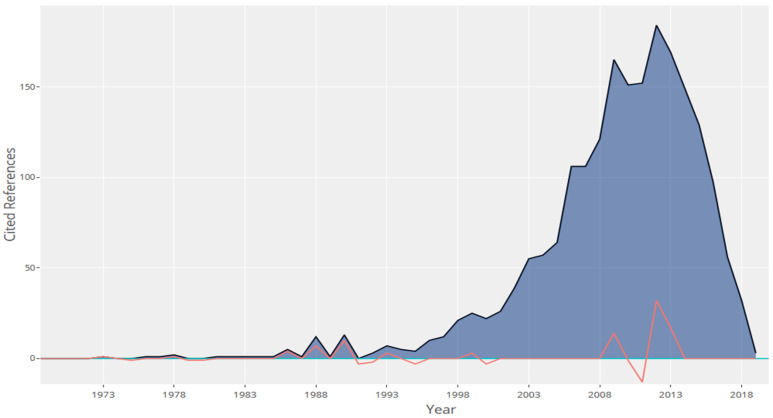
Reference publication year spectroscopy.

Figure 6 depicts the years of publication for all the references cited in the 80 selected works. There is a remarkable preponderance of current citations. The trend diminishes as of 2012, which is reasonable considering that our sample comprises papers published between 2015 and 2019 and, moreover, works cannot cite papers until they have been published. It is also worth considering that review processes for publishing are usually lengthy (between 1 and 2 years), which means that those from this period do not tend to be included in the cited references. 

### 4.2. RQ2:Where Has the VSM Methodology Been Applied since 2015? 

To answer this research question, we analyzed the healthcare settings and healthcare fields to which VSM has been applied since 2016. For healthcare settings, we identified the care levels (primary, secondary, tertiary) and the countries where the contributions found in the literature were from. We go on to provide details in the specific healthcare field where VSM has been applied.

**Figure 7:**
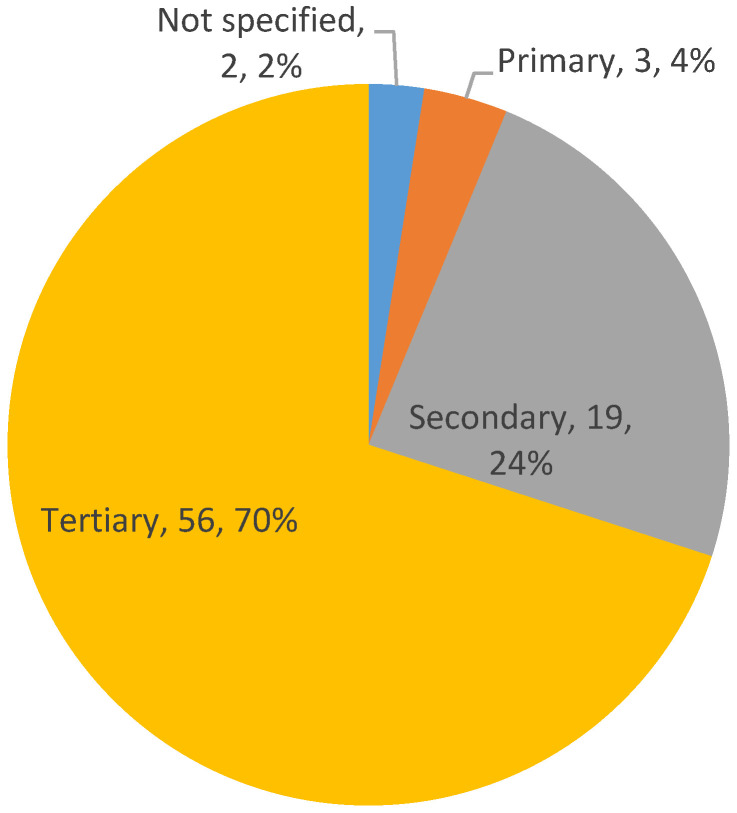
Distribution of health levels (Level; Frecuency; Percentage)

Figure 7 shows the distribution of the care levels to which the VSM methodology has been published since 2015. We can clearly see that such applications predominate in the tertiary sector; that is, applications in highly specialized medical care centers like hospitals. First, 56 applications appear in the tertiary sector, which represents 70% of the sample. Second, with 24% of all the works, VSM published applications appear in the secondary sector, composed mostly of clinics. The fact that very few applications appear in the primary sector stands out as there were only three, which represents 2% of the total.

**Figure 8:**
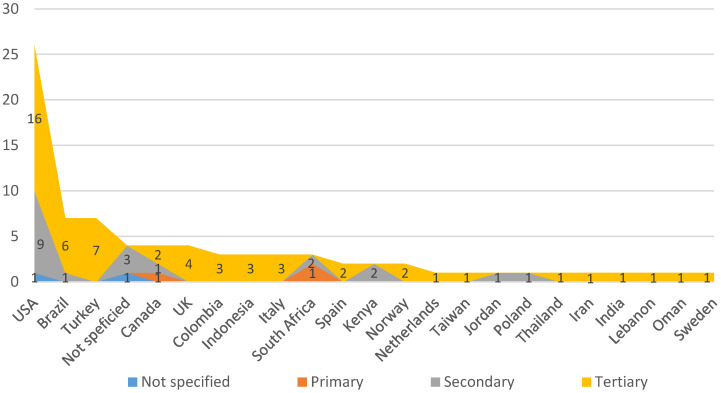
Number of applications per country.

Figure 8 offers details about which countries VSM published applications were performed in depending on care levels. Here, we see how the country with the most VSM applications is, once again, the USA, followed by Brazil and Turkey. Coherently with Figure 4, we find publications in accordance with their authors’ nationality. It is worth mentioning that some variations appear among all the articles in Figure 4 and Figure 8 because works appear in which their authors do not indicate the healthcare institution where they implement the VSM. According to Figure 8, those countries that report only one VSM application in the sample of articles tend to mention the tertiary sector. Only three countries can be seen (Kenya, Jordan and Poland) with exclusive applications at the secondary level. Applications in the tertiary sector tend to be combined with other levels.

Table 4 depicts the number of VSM applications for each healthcare field. More publications are found in emergency services (11), and usually at the tertiary level. The oncology and radiology fields are the next ones for number of publications (5), followed by laboratory (4) and Human Immunodeficiency Virus (HIV), Intensive Care Unit (ICU), pathology and pediatrics (3). The other healthcare fields only indicate two applications or one. Of the 11 VSM applications in emergency services, 5 appear in the general area, 2 in neurology, 1 in gynecology, another in radiology and 1 in maternal units. Of the five VSM applications in oncology, three are in the general area, a fourth is in gynecology and a fifth in radiation.

### 4.3. RQ3: What Is the Impact of VSM on Sustainability? 

**Figure 9:**
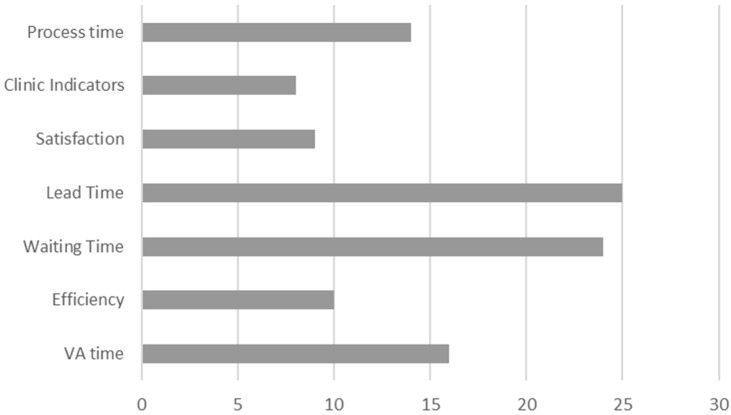
Main indicators identified as groups.

When we analyzed the articles, we found marked inconsistency in the indicators they used to identify/improve by means of the Value Stream Map. We grouped these indicators in the areas shown in Figure 9. Most articles are related to lead time: length of stay [78,80,81,143], in-the-door to out-the-door time [89], flow time [102,127,146], time of stay [90], stream time [114] and visiting time [111]. These indicators cannot be directly compared to one another as they deal with different actions or sections in healthcare. For instance, they go from processes in which patients go home for weeks or days to others in which patients are attended at hospitals within hours (e.g., emergency services). 

In relation to lead time, 24 articles deal with the problem of waiting times and 16 address the added-value/no added-value problem of processes, or the ratio and added-value. Lead time also improves if process times do, and 14 articles state some improvement in certain processes they study. 

Ten articles are about the efficiency or capacity of using resources, regardless of them being the human or physical resources employed in healthcare centers (percent usage, interventions/day, etc.).

The satisfaction problem for [79,84,89,99,108,129,150] or workers in health centers [86,107] comes across in nine articles. Nonetheless, it must be pointed out that in those cases when the lead time or waiting time has probably improved, it may be associated with patient satisfaction.

One interesting point is that the VSM technique can also help to improve certain medical operational indicators, such as duration of treatments or re-admission ratios [81,87,93,112,113,141,143,147].

Although it is not easy to group indicators in large sustainability areas (operational, environmental and social), we clearly observed that no indicator was directly linked with the environmental aspect, and the separation of indicators between social- and operational-type indicators in healthcare services was not clear. We identified worker satisfaction indicators as social sustainability. The other indicators found in the articles were associated with operational sustainability. However, as our work was conducted in the healthcare sector, reducing treatment costs (when processes improve) can serve to extend the population size that can access treatments, which can be taken as a social impact. 

### 4.4. RQ4: Is VSM Applied Alone or Combined with Other Lean Tools? 

**Figure 10:**
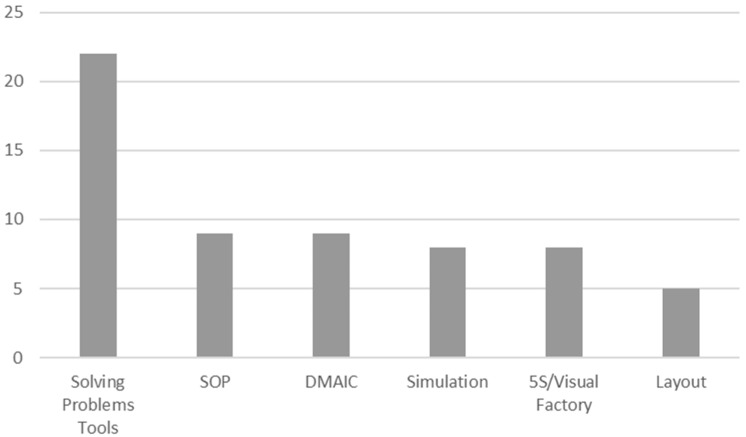
Number of improvement tools used with Value Stream Mapping (VSM).

The Value Stream Map is a tool employed to diagnose the present and to outline an action plan for an ideal future. Hence, using other improvement tools as a result of applying VSM is quite normal (Figure 10).

The most referenced improvement tools are those related to problem-solving methodologies. Some are a general set of tools, such as A3, but specific tools are normally employed, such as Ishikawa’s diagram or analyses of root causes. We did not include those papers that stated solving problems but then failed to specify which tools they used to do so. In relation to problem-solving, but in a different scope, we found that VSM formed part of tools like DMAIC/6-sigma (9 articles). The third most repeated tool is associated with processes standardization (SOP) (9 papers), which is not surprising because it is the basis of the Toyota Production System, a system that VSM encompasses. 

This figure does not show the papers that state using tools to identify waste (no added-value) (25 articles) because we assume that they all do, but do not explicitly state this. By definition, the VSM acts to identify those processes that add value and those that do not. It is also worth pointing out that quite a few of the studied articles employ diagrams of processes, although they are not indicated because, in most cases, they are identified as either VSM or variants of VSM.

The articles used more tools than those summarized in Figure 10 (poka yokes, mizusumashi, kaizen, Pareto analysis, PDCA, Supplier Input Process Output Customers SIPOC, heijunka, Single Minute Exchange of Die (SMED), etc.), which generally demonstrates that several lean tools are combined with VSM. 

### 4.5. RQ5: What Is the Most Common VSM Version in the Healthcare Sector Publications?

As defined in the methodology of Rother and Shook [11], VSM is a methodology to analyze and plan changes made toward a state that the team believes is ideal for a given setting. The model generally attempts to control the material flow so that the amount of product accumulated in the system is not too large and the product does not remain on the plant for too long. With healthcare systems, this would mean not having too many patients on waiting times. Nonetheless, other problems must be simultaneously dealt with on most occasions, which are identified when creating the Value Stream Map. The VSM methodology is divided into two main parts: creating the current state and the ideal state. To draw maps, a series of standardized symbols can be used to create a materials flow line (patients in the healthcare case), identify data movements with decision-making and create a timeline that distinguishes added-value activities from non-added-value ones. As the tool also serves to synchronize materials/patients, identifying waiting time is also deemed necessary. 

**Figure 11:**
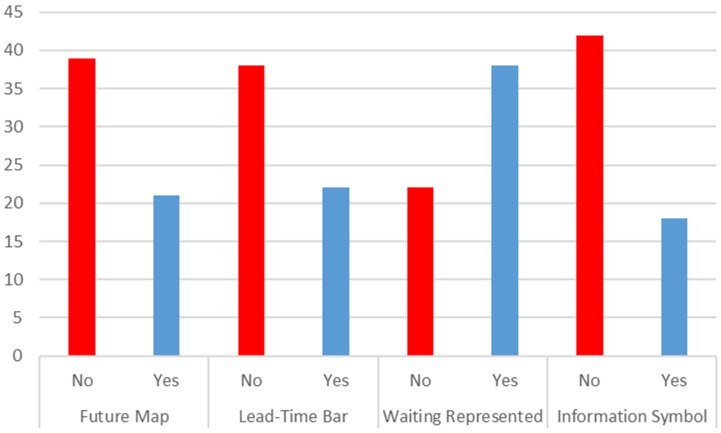
Standardization of the created map.

What we first point out is that of the 80 identified articles, 20 draw no kind of map despite them indicating one in the text. The following results were obtained in the rest (Figure 11).

To obtain the data that appear in Figure 11, we did not consider if they employed standardized symbols, but we verified if some differentiation existed in the sought aspects. We believe that waits can be identified if they meet any of these three conditions: if there is any specific type of symbol, if a clear gap on the map can be identified and if it is quantified with any number. Some examples of articles that respect all the VSM characteristics are References [91,127] while those that respect all the characteristics, except for information symbols, are References [82,115]

## 5. Discussion

Several studies [153,154,155] have identified that operations management can achieve improvements in healthcare areas. As the lean approach has been introduced in healthcare organizations, VSM and other tools related to process improvement have been incorporated into these organizations. Accordingly, the literature published about the VSM methodology in healthcare organizations has been growing, with certain ups and downs, over the years. The trend is similar to the publications on the lean approach in the healthcare sector [156,157]. It is a trend which is not exponential, as has happened with the publications of other operations management tools, such as the healthcare supply chain [156].

Taking into account the results shown in the articles reviewed in this study, the implementation of VSM is a potent tool to reduce waiting times for the patients. Also, the development of VSM improves patient flow and efficiency, similar to other tools related to lean healthcare [153,157,158,159]. The improvements have mainly come by way of reducing wasted time [160,161,162,163], and improving service quality, patient satisfaction [162,164,165] and safety [162,166,167]. The relation of these indicators to the areas of sustainability can be considered, with the environmental area remaining practically not covered by the published literature [168,169].

It is remarkable that we have not found any article with negative results or barely noticeable improvements. This is especially so considering that there are previous studies that indicate that the implementation of lean tools does not always provide the expected result [50,170]. Furthermore, the implementation in healthcare of lean tools, such as VSM, can be much more complicated than in a manufacturing context [170].

The VSM is commonly used in conjunction with other tools to differentiate value-added activities from activities with no value [154,171]. In terms of the maps drawn in the reviewed papers, it is common to find diagrams that are not exactly a VSM but are used to analyze processes. The tools most used with VSM in the analyzed papers are those associated with problem-solving. This aspect leads us to think that, once the problems with the flow of patients have been identified, they can be solved through these tools.

On the other hand, process mapping alone is not VSM methodology, at least in the model proposed by Rother [11] and widely followed in many papers in other sectors. If only the current state is analyzed, it does not fulfill the purpose of providing a clear roadmap for the organizations to improve. The future map was ignored in the majority of the articles examined for this study. This situation recalls the same issue shown in papers on other topics where the use of a part of the tools or steps in isolation does not guarantee the expected results from the application of the complete methodology [156,169].

The complexity of the healthcare system is generally considered an inhibitor of improvement initiatives [156,172,173]. However, none of the research analyzed has acknowledged this issue. Probably, when VSM is implemented in healthcare areas with similarities to manufacturing, such as laboratories or pharmacies, it tends to be more successful than when it is implemented in complex areas like emergency departments [156]. In the papers analyzed, experience in emergency departments predominates. This fact may be related to the internal organization of the health centers. These are organized based on departmental silos to extract maximum benefits of specialization. However, to improve patient flow, it is necessary to eliminate those silos and foster an integrated care process [156]. This is usually the case in emergency departments where almost all patients follow similar flows, and therefore, following the lean methodology would be more likely to achieve success.

All the VSM applications reported in the analyzed papers focus on improvements in a specific area, and VSM applications that cross departmental boundaries or incorporate other elements of the health chain are not shown. Such examples would include the link between primary care and medical specialties, or hospitals with external laboratories. This situation has been commented on in previous works [156,174], and it may mean that, instead of eliminating waste, in these cases, it is simply being transferred to another organizational unit. This may be an indicator that the focus of improvement through VSM is overly focused on local improvements and not on addressing substantial value chain improvements, as was proposed in Jones et al. [174]. It must be recognized that these types of transversal approaches are not profuse in the scientific literature of other sectors either.

Some authors suggest that establishing clear definitions for healthcare-related lean terminology would be interesting [169]. We consider as a future line of work not only clarifying terminology, but also establishing the appropriate indicators, their nomenclature and their calculation method in order to be able to share cases of success (and failure). In this way, the results could be made more extensible to other organizations.

Finally, the reported VSM implementations in several countries leave no doubt about the transferability of VSM in different contexts. However, many of the studies reviewed in which VSM is implemented have a lot of similarities, being single-case applications where the outcome to be improved is, mainly, time reduction [95,101,111]. The quality of these studies is diverse, and more quality research continues to be needed, not only in the design, but also in the reporting of it. In this line, some papers [82,127,139] can be presented as outstanding references in the development of the VSM methodology in healthcare.

## 6. Conclusions

The present scoping review described the potential size of available literature on VSM and healthcare, examined the extent, range and maturity of the research and determined research gaps in extant literature. Until 2015, very few research works had investigated using VSM in different sectors, and even fewer had done so specifically in healthcare services [10]. The present research work has shed some light on this point.

Integrating published knowledge is essential for a scientific field to develop [13]. On the one hand, it allows researchers to know all available current evidence and, on the other hand, they can know the limitations that affect available published research, as well as possible research niches that can be addressed by future research. Specifically, in relation to VSM, our research offers researchers a list of recent field works about the subject, including analyses of encountered pieces of evidence.

We can state that, although VSM started being generalized after Rother and Shook’s publication [11], the subject is still valid in healthcare settings 20 years later and many publications have appeared in recent years. Nevertheless, most published articles have referred to hospital contexts, which leaves primary care, which is critical for the general population’s health, outside the current research. This might be because the more complex services are, the more striking the improvements made. However, this gap should be bridged in the future.

The methodology is applicable to all areas in the healthcare sector, despite the inconsistencies found in how VSM has been used (selecting patient families, data collection, action plans, ways they are drawn). This clearly indicates that some kind of adaptation should be made from the industrial setting to the healthcare setting. For the results of VSM implementation to be more effective, it would be desirable for practitioners to have a robust and shared methodology, adapted to healthcare. For researchers, this standard format would allow outcomes to be verified and implementations to be validated so they could be later transferred to the healthcare industry with confirmed results. Nonetheless, as our results have checked and described, applying VSM seems to lead to major improvements in process indicators. 

The marked variety of countries where cases have been published is also noteworthy, as is the variety of analyzed healthcare systems (public, private and mixed managements). Another stressed point is that despite all the published articles presenting positive outcomes, we do not know if this is because there are no negative outcomes, or because they have not been published.

### Limitations and Future Research

This study presents some limitations that are worth noting. Firstly, the use of the WOS database only as a source of information. Despite WOS best covering the scientific journals in the health area, having included the SCOPUS database would have been helpful because it best covers the journals in the management and process improvement areas. Exploring databases with more professional type journals (like ABI and EBSCO) would have also been worthwhile, although such articles predominate in our results. Future research should explore these sources to see if they modify the results. The review scope could have also been extended to include laboratories as services susceptible to be analyzed with VSM. 

Another limitation was the lack of standardization in the indicators employed in the analyzed research papers. As this problem appeared in almost all the reviews conducted about performance in the management area [27,28], it would be interesting to attempt to somehow combine all the waiting time and total stay indicators of healthcare interventions. A wide range of terms appears (door-to-door, lead time, flow time, stream time), which may have their nuances and should be explained. Moreover, for healthcare management purposes, it would be very interesting to distinguish if waits are at the hospital or at home as this distinction was not found in the studied articles. 

As future research lines, it would be interesting to investigate the impact of implementing VSM in healthcare KPI. Almost all the publications mentioned improvement in process indicators. It is feasible to think that if processes improve, so would indicators of recovery, morbidity, etc., but this should be investigated in more detail to see if it would be confirmed. 

Regarding the use and drawing of Value Stream Maps, several problems were identified. First, the lack of symbols about information flows in 41 papers. This issue highlights the lack of attention to the patient scheduling problem when addressing the Value Stream Map. However, one of the fundamental objectives of the VSM is to address the problem of synchronizing the different activities so that the patient should not wait too long. Second, lack of standardization in symbols makes the different maps and indicators difficult to understand and compare. Third, there are not ideal state maps in 40 of the 60 papers. It means that a fundamental characteristic in the way to use maps in manufacturing settings (future state) might either prove complicated to adapt or may not be necessary in healthcare settings. Therefore, as a recommendation, we propose to unify the way to deal with process mapping. 

There are research questions still open, for example, which problems or difficulties have emerged while VSM was being drawn or after it was drawn? Which elements acted as mediators between VSM and the impact achieved by applying this technique? In what contexts did VSM work and where did it fail? Why? These issues can be dealt with in future research.

## Figures and Tables

**Table 1:** General bibliometric descriptions of the papers retrieved.

Description	Results
Documents	80
Article	71
Editorial material	1
Proceedings paper	8
Sources (Journals, Books, etc.)	67
Keywords Plus	164
Author’s Keywords	218
Period	2015–2019
Average citations per documents	4.784
Authors	408
Author Appearances	424
Authors of multi-authored documents	406
Single-authored documents	2
Documents per Author	0.196
Authors per Document	5.1
Collaboration Index	5.21

**Table 2:** Number of articles per author.

Authors	Articles
Cesarelli M	3
Improta G	3
Romano M	3
Balato G	2
Camgöz-Akdağ H	2
Cerfolio R. J	2
Neogi S	2
Russo M. A	2
Triassi M	2
Martínez P	2
Martínez J	2
Nuño P	2
Cavazos J	2

**Table 3:** Citations per references.

Paper	Total Citations	TC per Year
Hicks C, 2015, Int J Prod Econ [76]	43	7.167
Henrique D. B, 2016, Prod Plan Control [77]	38	7.600
Improta G, 2015, J Eval Clin Pract [78]	22	3.667
Tortorella G. L, 2017, Total Qual Manag Bus [75]	18	4.500
Duska L. R, 2015, Gynecol Oncol [79]	15	2.500
Dogan N. O, 2016, Total Qual Manag Bus [71]	14	2.800
Improta G, 2017, J Eval Clin Pract [80]	13	3.250
Cerfolio R. J, 2016, Ann Thorac Surg [81]	13	2.600
White B. A, 2017, West J Emerg Med [82]	12	3.000
Towbin A. J, 2017, Pediatr Radiol [83]	10	2.500
Sampalli T, 2015, Int J Health Policy [84]	10	1.667
Bal A, 2017, Int J Healthcare Man [85]	8	2.000
Sirvent J. M, 2016, Med Intensiva [86]	8	1.600
Swancutt D, 2017, BMC Health Serv Res [74]	7	1.750
Krupp N. L, 2017, J Asthma [87]	7	1.750
Haddad M. G, 2016, Eng Manag J [88]	7	1.400
Robinson F. G, 2016, J Dent Educ [89]	7	1.400
Cheung Y. Y, 2016, Radiographics [90]	7	1.400

**Table 4:** Articles per healthcare field.

Healthcare Field	Articles	No.
Emergency	[74,82,85,91,92,93,94,95,96,97,98]	11
Oncology	[77,79,99,100,101]	5
Radiology	[83,90,102,103,104]	5
Laboratory	[105,106,107,108]	4
HIV	[109,110,111]	3
Intensive Care Unit	[86,112,113]	3
Pathology	[114,115,116]	3
Pediatrics	[87,117,118]	3
Cardiothoracic Surgery and Anesthesiology	[81,119]	2
Chronic Service	[84,120]	2
Maternity Unit	[121,122]	2
Medicine dispensation	[123,124]	2
Orthopedics and Traumatology	[78,80]	2
Outpatient Services	[125,126]	2
Physical Therapy and Rehabilitation	[71,127]	2
Sterilization	[75,128]	2
Surgical Unit	[129,130]	2
Transplants	[131,132]	2
Anesthesia	[133]	1
Audiology	[134]	1
Cardiology	[135]	1
Dental	[89]	1
Endocrine	[136]	1
Endoscopy	[76]	1
Gynecology	[137]	1
Glaucoma	[138]	1
Hemophilia	[139]	1
Orthopedics	[140]	1
Otolaryngology	[141]	1
Outpatient Internal Medicine	[142]	1
Trauma	[143]	1
**Others**	**Articles**	**No.**
Not specified	[144,145,146,147]	4
Administrative	[148,149]	2
Clinical Publications	[150]	1
Hospital Admissions	[88]	1
Internal saline process	[151]	1
Food services	[152]	1

## Data Availability

Request to the correspondence author.

## References

[1] De Souza Gomes dos Santos A.C., da Cunha Reis A., de Dos S.G., Santos C., Ferreria I.L., Figueiredo L.A. (2020). The first evidence about conceptual vs analytical lean healthcare research studies. J. Health Organ. Manag..

[2] Dahlgaard J.J., Pettersen J., Dahlgaard-Park S.M. (2011). Quality and lean health care: A system for assessing and improving the health of healthcare organisations. Total Qual. Manag. Bus. Excell..

[3] Marin-Garcia J.A., Bonavia T., Chiaberge M. (2011). Strategic Priorities and Lean Manufacturing Practices in Automotive Suppliers. Ten Years After. New Trends and Developments in Automotive Engineering.

[4] Marin-Garcia J.A., Carneiro P. (2010). Desarrollo y validación de un modelo multidimensional de la producción ajustada. Intang. Cap..

[5] Marin-Garcia J.A., Carneiro P., Miralles C., Mejia G., Velasco N. (2012). Effect of Lean Manufacturing practices on non-financial performance results: Empirical study in Spanish sheltered work centers. Production Systems and Supply Chain Management in Emerging Countries: Best Practices.

[6] Scott G. (2001). Customer Satisfaction: Six Strategies for Continuous Improvement. J. Healthc. Manag..

[7] Marin-Garcia J.A., Mateo Martínez R. (2013). Barreras y facilitadores de la implantación del TPM. Intang. Cap..

[8] Vidal-Carreras P.I., Garcia-Sabater J.J., Marin-Garcia J.A., Garcia-Sabater J.P., Framinan J.M., Gonzalez P.P., Artiba A. (2015). Value Stream Mapping on Healthcare. 2015 International Conference on Industrial Engineering and Systems Management (IESM).

[9] Coetzee R., van der Merwe K., van Dyk L. (2016). Lean implementation strategies: How are the Toyota Way principles addressed?. S. Afr. J. Ind. Eng..

[10] Shou W., Wang J., Wu P., Wang X., Chong H.-Y. (2017). A cross-sector review on the use of value stream mapping. Int. J. Prod. Res..

[11] Rother M., Shook J. (1998). Learning to See: Value Stream Mapping to Create Value and Eliminate Muda.

[12] Muñoz-Villamizar A., Santos J., Garcia-Sabater J.J., Lleo A., Grau P. (2019). Green value stream mapping approach to improving productivity and environmental performance. Int. J. Product. Perform. Manag..

[13] Borenstein M., Hedges L.V., Higgins J.P.T., Rothstein H.R. (2009). Introduction to Meta-analysis.

[14] Nowak M., Pfaff H., Karbach U. (2017). Does Value Stream Mapping affect the structure, process, and outcome quality in care facilities? A systematic review. Syst. Rev..

[15] Dinis-Carvalho J., Guimaraes L., Sousa R.M., Leao C.P. (2019). Waste identification diagram and value stream mapping: A comparative analysis. Int. J. Lean Six Sigma.

[16] Hines P., Rich N. (1997). The seven value stream mapping tools. Int. J. Oper. Prod. Manag..

[17] Lucherini F., Rapaccini M. (2017). Exploring the impact of lean manufacturing on flexibility in SMEs. J. Ind. Eng. Manag..

[18] Bevilacqua M., Ciarapica F.E., Germani M., Mazzuto G., Paciarotti C. (2014). Relation of project managers’ personality and project performance: An approach based on value stream mapping. J. Ind. Eng. Manag..

[19] Tapping D. (2007). La Nueva Guia Lean de Bolsillo (Produccion Lean). Herramientas Para Eliminar el Desperdicio.

[20] Tapping D., Luyster T., Shuker T. (2002). Value Stream Management Eight Steps to Planning, Mapping, and Sustaining Lean Improvements.

[21] Tapping D., Shuker T. (2003). Value Stream Management for the Lean Office.

[22] Cringles M.C. (2002). Developing an integrated care pathway to manage cancer pain across primary, secondary and tertiary care. Int. J. Palliat. Nurs..

[23] Grumbach K., Bodenheimer T. (1995). The Organization of Health Care. JAMA.

[24] Ahluwalia S.C., Damberg C.L., Silverman M., Motala A., Shekelle P.G. (2017). What Defines a High-Performing Health Care Delivery System: A Systematic Review. Jt. Comm. J. Qual. Patient Saf..

[25] Sanchez-Ruiz L., Blanco B., Marin-Garcia J.A., Diez-Busto E. (2020). Scoping Review of Kaizen and Green Practices: State of the Art and Future Directions. Int. J. Environ. Res. Public Health.

[26] Urdan M.S., Luoma P. (2020). Designing Effective Sustainability Assignments: How and Why Definitions of Sustainability Impact Assignments and Learning Outcomes. J. Manag. Educ..

[27] Gaikwad L., Sunnapwar V. (2020). An integrated Lean, Green and Six Sigma strategies: A systematic literature review and directions for future research. TQM J..

[28] Lorenzon dos Santos D., Giglio R., Helleno A.L., Campos L.M.S. (2019). Environmental aspects in VSM: A study about barriers and drivers. Prod. Plan. Control.

[29] Helleno A.L., de Moraes A.J.I., Simon A.T. (2017). Integrating sustainability indicators and Lean Manufacturing to assess manufacturing processes: Application case studies in Brazilian industry. J. Clean. Prod..

[30] Farias L.M.S., Santos L.C., Gohr C.F., de Oliveira L.C., da Silva Amorim M.H. (2019). Criteria and practices for lean and green performance assessment: Systematic review and conceptual framework. J. Clean. Prod..

[31] Antony J., Sunder M V., Sreedharan R., Chakraborty A., Gunasekaran A. (2019). A systematic review of Lean in healthcare: A global prospective. Int. J. Qual. Reliab. Manag..

[32] Abdallah A.B., Alkhaldi R.Z. (2019). Lean bundles in health care: A scoping review. J. Health Organ. Manag..

[33] Podgorodnichenko N., Akmal A., Edgar F., Everett A.M. (2020). Sustainable HRM: Toward addressing diverse employee roles. Empl. Relat..

[34] Marin-Garcia J.A., Bonavia T., Losilla J.-M. (2020). Changes in the Association between European Workers’ Employment Conditions and Employee Well-being in 2005, 2010 and 2015. Int. J. Environ. Res. Public Health.

[35] Saunders M., Lewis P., Thornhill A. (2016). Research Methods for Business Students, 7/e.

[36] Makwana A.D., Patange G.S. (2019). A methodical literature review on application of Lean & Six Sigma in various industries. Aust. J. Mech. Eng..

[37] Romero L.F., Arce A. (2017). Applying Value Stream Mapping in Manufacturing: A Systematic Literature Review. IFAC-PapersOnLine.

[38] Singh B., Garg S.K., Sharma S.K. (2011). Value stream mapping: Literature review and implications for Indian industry. Int. J. Adv. Manuf. Technol..

[39] Augusto B.P., Tortorella G.L. (2019). Literature review on lean healthcare implementation: Assessment methods and practices. Int. J. Serv. Oper. Manag..

[40] Syltevik S., Karamperidis S., Antony J., Taheri B. (2018). Lean for airport services: A systematic literature review and agenda for future research. Int. J. Qual. Reliab. Manag..

[41] Rafique M.Z., Ab Rahman M.N., Saibani N., Arsad N. (2019). A systematic review of lean implementation approaches: A proposed technology combined lean implementation framework. Total Qual. Manag. Bus. Excell..

[42] Poksinska B. (2010). The current state of lean implementation in health care: Literature review. Qual. Manag. Health Care.

[43] Gitlow H., Zuo Q., Ullmann S.G., Zambrana D., Campo R.E., Lubarsky D., Birnbach D.J. (2013). The causes of never events in hospitals. Int. J. Lean Six Sigma.

[44] Heinzen M., Mettler S., Coradi A., Boutellier R. (2015). A new application of value-stream mapping in new drug development: A case study within Novartis. Drug Discov. Today.

[45] Marin-Garcia J.A., Garcia-Sabater J.J., Maheut J. (2018). Protocol: Action planning for action research about kaizen in public organizations. The case of higher education. WPOM-Work. Pap. Oper. Manag..

[46] Alrashed I.A., Kang P.S. (2017). Applying lean principles to health economics transactional flow process to improve the healthcare delivery. Proceedings of the 2017 IEEE International Conference on Industrial Engineering and Engineering Management (IEEM).

[47] Bhat S., Antony J., Gijo E.V., Cudney E.A. (2019). Lean Six Sigma for the healthcare sector: A multiple case study analysis from the Indian context. Int. J. Qual. Reliab. Manag..

[48] Costa L.B.M., Filho M.G., Rentes A.F., Bertani T.M., Mardegan R. (2017). Lean healthcare in developing countries: Evidence from Brazilian hospitals. Int. J. Health Plann. Manag..

[49] Bucci S., De Belvis A.G., Marventano S., De Leva A.C., Tanzariello M., Specchia M.L., Ricciardi W., Franceschi F. (2016). Emergency Department crowding and hospital bed shortage: Is Lean a smart answer? A systematic review. Eur. Rev. Med. Pharmacol. Sci..

[50] Moraros J., Lemstra M., Nwankwo C. (2016). Lean interventions in healthcare: Do they actually work? A systematic literature review. Int. J. Qual. Heal. Care.

[51] Cobo M.J., López-Herrera A.G., Herrera-Viedma E., Herrera F. (2012). SciMAT: A new science mapping analysis software tool. J. Am. Soc. Inf. Sci. Technol..

[52] Aria M. (2017). bibliometrix: An R-tool for comprehensive science mapping analysis. J. Informetr..

[53] Aguinis H., Ramani R.S., Alabduljader N. (2020). Best-Practice Recommendations for Producers, Evaluators, and Users of Methodological Literature Reviews. Organ. Res. Methods.

[54] Andreu Andres M.A., Garcia-Carbonell A., González-Ladrón-de-Guevara F., Watts F. (2018). Contrasting innovation competence FINCODA model in software engineering: Narrative review. J. Ind. Eng. Manag..

[55] Fadahunsi K.P., Akinlua J.T., O’Connor S., Wark P.A., Gallagher J., Carroll C., Majeed A., O’Donoghue J. (2019). Protocol for a systematic review and qualitative synthesis of information quality frameworks in eHealth. BMJ Open.

[56] Marin-Garcia J.A., Alfalla-Luque R. (2019). Protocol: How to deal with Partial Least Squares (PLS) research in Operations Management. A guide for sending papers to academic journals. WPOM-Work. Pap. Oper. Manag..

[57] Marin-Garcia J.A., Betancour E., Giraldo-OMeara M. (2018). Protocol: Literature review on the psychometric properties of the short versions of the scales of social desirability in the answers to competency self-assessment questionnaires. WPOM-Work. Pap. Oper. Manag..

[58] Martínez Jurado P.J., Moyano Fuentes J. (2017). Aprendiendo a Enseñar Lean Management mediante Juegos: Revisión Sistemática de la Literatura Learning to Teach Lean Management through Games: Systematic Literature Review. WPOM-Work. Pap. Oper. Manag..

[59] Medina-López C., Marin-Garcia J.A., Alfalla Luque R. (2010). Una propuesta metodológica para la realización de búsquedas sistemáticas de bibliografía (A methodological proposal for the systematic literature review). WPOM.

[60] Marin-Garcia J.A., Martinez-Tomas J. (2019). Protocol: What does the wage structure depend on? Evidence from the INE salary national survey (pilot study with 2006 data). WPOM-Work. Pap. Oper. Manag..

[61] Marin-Garcia J.A., Vidal-Carreras P.I., Garcia Sabater J.J., Escribano-Martinez J. (2019). Protocol: Value Stream Maping in Healthcare. A systematic literature review. WPOM-Work. Pap. Oper. Manag..

[62] Aloini D., Cannavacciuolo L., Gitto S., Lettieri E., Malighetti P., Visintin F. (2018). Evidence-Based Management for Performance Improvement in HealthCare. Manag. Decis..

[63] Reijula J., Nevala N., Lahtinen M., Ruohomäki V., Reijula K. (2014). Lean design improves both health-care facilities and processes: A literature review. Intell. Build. Int..

[64] Gonzalez-Aleu F., Van Aken E.M., Cross J., Glover W.J. (2018). Continuous improvement project within Kaizen: Critical success factors in hospitals. TQM J..

[65] Losilla J.-M., Oliveras I., Marin-Garcia J.A., Vives J. (2018). Three risk of bias tools lead to opposite conclusions in observational research synthesis. J. Clin. Epidemiol..

[66] Marin-Garcia J.A., Martinez Tomas J. (2016). Deconstructing AMO framework: A systematic review. Intang. Cap..

[67] Sanchez L., Blanco B. (2014). Three decades of continuous improvement. Total Qual. Manag. Bus. Excell..

[68] Sanchez-Ruiz L., Marin-Garcia J.A., Blanco B. (2018). Protocol: A Meta-Review on continuous improvement to know the state of this research field. WPOM-Working Pap. Oper. Manag..

[69] Knight C., Patterson M., Dawson J. (2019). Work engagement interventions can be effective: A systematic review. Eur. J. Work Organ. Psychol..

[70] Lummus R.R., Vokurka R.J., Rodeghiero B. (2006). Improving quality through value stream mapping: A case study of a physician’s clinic. Total Qual. Manag. Bus. Excell..

[71] Dogan N.O., Unutulmaz O. (2016). Lean production in healthcare: A simulation-based value stream mapping in the physical therapy and rehabilitation department of a public hospital. Total Qual. Manag. Bus. Excell..

[72] Vandborg M.P., Edwards K., Kragstrup J., Vedsted P., Hansen D.G., Mogensen O. (2012). A New Method for Analyzing Diagnostic Delay in Gynecological Cancer. Int. J. Gynecol. Cancer.

[73] Wulff Barreiro E. (2007). El uso del software HistCite para identificar artículos significativos en búsquedas por materias en la Web of Science. Doc. las Ciencias la Inf..

[74] Swancutt D., Joel-Edgar S., Allen M., Thomas D., Brant H., Benger J., Byng R., Pinkney J. (2017). Not all waits are equal: An exploratory investigation of emergency care patient pathways. BMC Health Serv. Res..

[75] Tortorella G.L., Fogliatto F.S., Anzanello M., Marodin G.A., Garcia M., Reis Esteves R. (2017). Making the value flow: Application of value stream mapping in a Brazilian public healthcare organisation. Total Qual. Manag. Bus. Excell..

[76] Hicks C., McGovern T., Prior G., Smith I. (2015). Applying lean principles to the design of healthcare facilities. Int. J. Prod. Econ..

[77] Henrique D.B., Rentes A.F., Godinho Filho M., Esposto K.F. (2016). A new value stream mapping approach for healthcare environments. Prod. Plan. Control.

[78] Improta G., Balato G., Romano M., Carpentieri F., Bifulco P., Alessandro Russo M., Rosa D., Triassi M., Cesarelli M. (2015). Lean Six Sigma: A new approach to the management of patients undergoing prosthetic hip replacement surgery. J. Eval. Clin. Pract..

[79] Duska L.R., Mueller J., Lothamer H., Pelkofski E.B., Novicoff W.M. (2015). Lean methodology improves efficiency in outpatient academic Gynecologic Oncology clinics. Gynecol. Oncol..

[80] Improta G., Balato G., Romano M., Ponsiglione A.M., Raiola E., Russo M.A., Cuccaro P., Santillo L.C., Cesarelli M. (2017). Improving performances of the knee replacement surgery process by applying DMAIC principles. J. Eval. Clin. Pract..

[81] Cerfolio R.J., Steenwyk B.L., Watson C., Sparrow J., Belopolsky V., Townsley M., Lyerly R., Downing M., Bryant A., Gurley W.Q. (2016). Decreasing the Preincision Time for Pulmonary Lobectomy: The Process of Lean and Value Stream Mapping. Ann. Thorac. Surg..

[82] White B.A., Yun B.J., Lev M.H., Raja A.S. (2017). Applying Systems Engineering Reduces Radiology Transport Cycle Times in the Emergency Department. West. J. Emerg. Med..

[83] Towbin A.J., Perry L.A., Larson D.B. (2017). Improving efficiency in the radiology department. Pediatr. Radiol..

[84] Sampalli T., Desy M., Dhir M., Edwards L., Dickson R. (2015). Improving wait times to care for individuals with multimorbidities and complex conditions using value stream mapping. Int. J. Heal. Policy Manag..

[85] Bal A., Ceylan C., Taçoğlu C. (2017). Using value stream mapping and discrete event simulation to improve efficiency of emergency departments. Int. J. Healthc. Manag..

[86] Sirvent J.M., Gil M., Alvarez T., Martin S., Vila N., Colomer M., March E., Loma-Osorio P., Metje T. (2016). Técnicas «Lean» para la mejora del flujo de los pacientes críticos de una región sanitaria con epicentro en el servicio de medicina intensiva de un hospital de referencia. Med. Intensiva.

[87] Krupp N.L., Fiscus C., Webb R., Webber E.C., Stanley T., Pettit R., Davis A., Hollingsworth J., Bagley D., McCaskey M. (2017). Multifaceted quality improvement initiative to decrease pediatric asthma readmissions. J. Asthma.

[88] Haddad M.G., Zouein P.P., Salem J., Otayek R. (2016). Case Study of Lean in Hospital Admissions to Inspire Culture Change. Eng. Manag. J..

[89] Robinson F.G., Cunningham L.L., Turner S.P., Lindroth J., Ray D., Khan T., Yates A. (2016). Improving a Dental School’s Clinic Operations Using Lean Process Improvement. J. Dent. Educ..

[90] Cheung Y.Y., Goodman E.M., Osunkoya T.O. (2016). No More Waits and Delays: Streamlining Workflow to Decrease Patient Time of Stay for Image-guided Musculoskeletal Procedures. RadioGraphics.

[91] Martínez P., Martínez J.L., Cavazos J., Nuño J.P. (2016). Mejora en el tiempo de atención al paciente en una Unidad de urgencias por medio de Lean Manufacturing. Nov. Sci..

[92] Martínez P., Martínez J., Nuño P., Cavazos J. (2016). Mejora en el tiempo de atención al paciente en una unidad de urgencias gineco-obstétricas mediante la aplicación de Lean Manufacturing. Rev. Lasallista Investig..

[93] Marisdina S., Junadi P. (2018). Utilizing Lean Management Techniques to Improve Head CT Scan Turnaround Time of Ischemic Stroke Patients in the Emergency Department of Dr. Mohammad Hoesin Hospital, Palembang. KnE Life Sci..

[94] Sánchez M., Suárez M., Asenjo M., Bragulat E. (2018). Improvement of emergency department patient flow using lean thinking. Int. J. Qual. Health Care J. Int. Soc. Qual. Health Care.

[95] Firman F., Koentjoro T., Widodo K.H., Utarini A. (2019). The effect of lean six sigma toward maternal emergency lead time in penembahan senopati hospital, bantul, yogyakarta. BALI Med. J..

[96] Improta G., Romano M., Di Cicco M.V., Ferraro A., Borrelli A., Verdoliva C., Triassi M., Cesarelli M. (2018). Lean thinking to improve emergency department throughput at AORN Cardarelli hospital. BMC Health Serv. Res..

[97] Damle A., Andrew N., Kaur S., Orquiola A., Alavi K., Steele S.R., Maykel J. (2016). Elimination of waste: Creation of a successful Lean colonoscopy program at an academic medical center. Surg. Endosc..

[98] Lisiecka-Biełanowicz M., Biechowska D., Brzozowski S., Hermanowski T. (2018). Mapping process of the stroke treatment at the Institute of Psychiatry and Neurology in Warsaw. Postępy Psychiatr. Neurol..

[99] Al Hroub A., Obaid A., Yaseen R., El-Aqoul A., Zghool N., Abu-Khudair H., Al Kakani D., Alloubani A. (2019). Improving the Workflow Efficiency of an Outpatient Pain Clinic at a Specialized Oncology Center by Implementing Lean Principles. Asia-Pacific J. Oncol. Nurs..

[100] Al-Balushi M.M., Al-Mandhari Z. (2018). Implementing Lean Management Techniques at a Radiation Oncology Department. Sultan Qaboos Univ. Med. J..

[101] Gjolaj L.N., Campos G.G., Olier-Pino A.I., Fernandez G.L. (2016). Delivering Patient Value by Using Process Improvement Tools to Decrease Patient Wait Time in an Outpatient Oncology Infusion Unit. J. Oncol. Pract..

[102] Akdag H.C., Cantürk N.Z. (2017). Improvement of Breast Cancer Patient Pathway Using EUSOMA Standards and European Guidelines. Chirurgia.

[103] Camgoz-Akdag H., Beldek T., Konyalioglu A.K. (2018). Process improvement in a radiology department with value stream mapping and its linkage to industry 4.0. Iioab J..

[104] Camgöz-Akdağ H., Çalişkan E., Toma S. (2017). Lean process design for a radiology department. Bus. Process Manag. J..

[105] de Oliveira K.B., dos Santos E.F., Junior L.V.G., Duffy V.G., Lightner N. (2017). Lean Healthcare as a Tool for Improvement: A Case Study in a Clinical Laboratory. Advances in Human Factors and Ergonomics in Healthcare.

[106] Alkher M., Radosevic M., Beker I., Cabarkapa V., Toljaga-nikolic D., Caric M., Moraca S. (2019). Case Study of Healthcare Organization Improvement with Lean Concept. Teh. Vjesn. Tech. Gaz..

[107] Roy A., Colpitts J., Becker K., Brewer J., van Lutterveld R. (2018). Improving efficiency in neuroimaging research through application of Lean principles. PLoS ONE.

[108] Gupta S., Kapil S., Sharma M. (2018). Improvement of laboratory turnaround time using lean methodology. Int. J. Health Care Qual. Assur..

[109] Monroe-Wise A., Reisner E., Sherr K., Ojakaa D., Mbau L., Kisia P., Muhula S., Farquhar C. (2017). Using lean manufacturing principles to evaluate wait times for HIV-positive patients in an urban clinic in Kenya. Int. J. STD AIDS.

[110] Hoffmann C.J., Milovanovic M., Kinghorn A., Kim H.-Y., Motlhaoleng K., Martinson N.A., Variava E. (2018). Value stream mapping to characterize value and waste associated with accessing HIV care in South Africa. PLoS ONE.

[111] Mabuto T., Hansoti B., Kerrigan D., Mshweshwe-Pakela N., Kubeka G., Charalambous S., Hoffmann C. (2019). HIV testing services in healthcare facilities in South Africa: A missed opportunity. J. Int. AIDS Soc..

[112] van der Sluijs A.F., van Slobbe-Bijlsma E.R., Goossens A., Vlaar A.P., Dongelmans D.A. (2019). Reducing errors in the administration of medication with infusion pumps in the intensive care department: A lean approach. SAGE Open Med..

[113] Ersson A., Beckman A., Jarl J., Borell J. (2018). Effects of a multifaceted intervention QI program to improve ICU performance. BMC Health Serv. Res..

[114] Durur F., Akbulut Y. (2019). Lean methodology for pathology laboratories: A case study from a public hospital. Turkish J. Pathol..

[115] Sugianto J.Z., Stewart B., Ambruzs J.M., Arista A., Park J.Y., Cope-Yokoyama S., Luu H.S. (2015). Applying the Principles of Lean Production to Gastrointestinal Biopsy Handling: From the Factory Floor to the Anatomic Pathology Laboratory. Lab. Med..

[116] Cromwell S., Chiasson D.A., Cassidy D., Somers G.R. (2018). Improving Autopsy Report Turnaround Times by Implementing Lean Management Principles. Pediatr. Dev. Pathol..

[117] Ankrum A.L., Neogi S., Morckel M.A., Wilhite A.W., Li Z., Schaffzin J.K. (2019). Reduced isolation room turnover time using Lean methodology. Infect. Control Hosp. Epidemiol..

[118] Fields E., Neogi S., Schoettker P.J., Lail J. (2018). Using Lean methodologies to streamline processing of requests for durable medical equipment and supplies for children with complex conditions. Healthcare.

[119] Cerfolio R.J., Ferrari-Light D., Ren-Fielding C., Fielding G., Perry N., Rabinovich A., Saraceni M., Fitzpatrick M., Jain S., Pachter H.L. (2019). Improving Operating Room Turnover Time in a New York City Academic Hospital via Lean. Ann. Thorac. Surg..

[120] Lebina L., Alaba O., Ringane A., Hlongwane K., Pule P., Oni T., Kawonga M. (2019). Process evaluation of implementation fidelity of the integrated chronic disease management model in two districts, South Africa. BMC Health Serv Res..

[121] Ramaswamy R., Rothschild C., Alabi F., Wachira E., Muigai F., Pearson N. (2017). Using Value Stream Mapping to improve quality of care in low-resource facility settings. Int. J. Qual. Health Care.

[122] Smith I. (2016). Operationalising the Lean principles in maternity service design using 3P methodology. BMJ Qual. Improv. Rep..

[123] Da Costa D.G., Pasin S.S., de Magalhães A.M.M., de Moura G.M.S.S., Rosso C.B., Saurin T.A. (2018). Análise do preparo e administração de medicamentos no contexto hospitalar com base no pensamento Lean. Esc. Anna Nery Rev. Enferm..

[124] Pazeti M., Calache L., Duffy V.G., Lightner N. (2017). Application of Lean Six Sigma Concepts to Medicine Dispensation of Public Health Centers. Advances in Human Factors and Ergonomics in Healthcare.

[125] Johannessen K.A., Alexandersen N. (2018). Improving accessibility for outpatients in specialist clinics: Reducing long waiting times and waiting lists with a simple analytic approach. BMC Health Serv. Res..

[126] Sayyida G., Fahma F., Iftadi I. (2018). Process Improvement in Outpatient Installation RSUD dr. Soediran Mangun Sumarso Using Lean Hospital Approach. IOP Conf. Ser. Mater. Sci. Eng..

[127] Deniz N., Özçelik F. (2018). Improving healthcare service processes by lean thinking. Pamukkale Univ. J. Eng. Sci..

[128] Henrique H.A., Carolina B.A., Priscilla L., Lhotska L., Sukupova L., Lackovic I., Ibbott G. (2018). Application of Engineering Concepts in the Sterile Processing Department. World Congress on Medical Physics and Biomedical Engineering.

[129] Wannemuehler T.J., Elghouche A.N., Kokoska M.S., Deig C.R., Matt B.H. (2015). Impact of Lean on surgical instrument reduction: Less is more. Laryngoscope.

[130] Hung S.-H., Wang P.-C., Lin H.-C., Chen H.-Y., Su C.-T. (2015). Integration of Value Stream Map and Healthcare Failure Mode and Effect Analysis into Six Sigma Methodology to Improve Process of Surgical Specimen Handling. J. Healthc. Eng..

[131] Lot L.T., Sarantopoulos A., Min L.L., Perales S.R., de Fatima Santana Ferreira Boin I., de Ataide E.C. (2018). Using Lean tools to reduce patient waiting time. Leadersh. Health Serv..

[132] Barakauskas V.E., Bradshaw T.A., Smith L.D., Lehman C.M., Johnson-Davis K.L. (2016). Process Optimization to Improve Immunosuppressant Drug Testing Turnaround Time. Am. J. Clin. Pathol..

[133] Khodambashi S. (2015). Alignment of an intra-operating management process to a health information system: A Lean analysis approach. Pers. UBIQUITOUS Comput..

[134] Huddle M.G., Tirabassi A., Turner L., Lee E., Ries K., Lin S.Y. (2016). Application of Lean Sigma to the Audiology Clinic at a Large Academic Center. Otolaryngol. NECK Surg..

[135] Antosz K., Stadnicka D., Ratnayake R.M.C. (2016). Use of lean management philosophy in health sector: A VSM based case study. Proceedings of the 2016 IEEE International Conference on Industrial Engineering and Engineering Management (IEEM).

[136] Mascarella M.A., Lahrichi N., Cloutier F., Kleiman S., Payne R.J., Rosenberg L. (2016). High efficiency endocrine operation protocol: From design to implementation. Surgery.

[137] Anisi S., Marzban S., Zarei E., Sepehri M.M. (2017). Identifying process improvement opportunities in gynecology clinic by value stream mapping. Iioab J..

[138] Newman-Casey P.A., Musser J.A., Niziol L.M., Heisler M.M., Kamat S.S., Shah M.M., Patel N., Cohn A.M. (2019). Integrating Patient Education Into the Glaucoma Clinical Encounter. J. Glaucoma.

[139] Drayton Jackson M., Bartman T., McGinniss J., Widener P., Dunn A.L. (2019). Optimizing patient flow in a multidisciplinary haemophilia clinic using quality improvement methodology. Haemophilia.

[140] Gleich S.J., Nemergut M.E., Stans A.A., Haile D.T., Feigal S.A., Heinrich A.L., Bosley C.L., Tripathi S. (2016). Improvement in patient transfer process from the operating room to the PICU using a Lean and Six Sigma--based quality improvement project. Hosp. Pediatr..

[141] Gray M.L., Chen S., Kinberg E., Colley P., Malkin B.D. (2019). Using Lean to Improve Patient Safety and Resource Utilization After Pediatric Adenotonsillectomy. J. Patient Saf..

[142] Ortíz-Barrios M.A., Escorcia-Caballero J.P., Sánchez-Sánchez F., De Felice F., Petrillo A. (2017). Efficiency Analysis of Integrated Public Hospital Networks in Outpatient Internal Medicine. J. Med. Syst..

[143] Ajdari A., Boyle L.N., Kannan N., Rowhani-Rahbar A., Wang J., Mink R., Ries B., Wainwright M., Groner J.I., Bell M.J. (2017). Examining Emergency Department Treatment Processes in Severe Pediatric Traumatic Brain Injury. J. Healthc. Qual..

[144] Özcan-Top Ö., McCaffery F. (2018). A hybrid assessment approach for medical device software development companies. J. Softw. Evol. Process.

[145] Schmidt S., Shay L.A., Saygin C., Wan H., Schulz K., Clark R.A., Shireman P.K. (2018). Improving pilot project application and review processes: A novel application of lean six sigma in translational science. J. Clin. Transl. Sci..

[146] Lin J.-H., Chiu M.-C., Chen C.H., Trappey A.C., Peruzzini M., Stjepandic J., Wognum N. (2017). A Mathematical Model to Evaluate and Improve Lean Management of Healthcare System: A Case Study of Health Examination Center. Transdisciplinary Engineering: A Paradigm Shift.

[147] Afsar-manesh N., Lonowski S., Namavar A.A. (2017). Leveraging lean principles in creating a comprehensive quality program: The UCLA health readmission reduction initiative. Healthcare.

[148] Martinez D.A., Tsalatsanis A., Yalcin A., Zayas-Castro J.L., Djulbegovic B. (2016). Activating clinical trials: A process improvement approach. Trials.

[149] MacDonald S.L., Joseph P.L., Cavaliere I.J., Bayley M.T., Lo A. (2018). Optimising the mandatory reporting process for drivers admitted to an inpatient stroke rehabilitation unit. BMJ Open Qual..

[150] Valdez M.M., Liwanag M., Mount C., Rodriguez R., Avalos-Reyes E., Smith A., Collette D., Starsiak M., Green R. (2018). Utilizing Lean Six Sigma Methodology to Improve the Authored Works Command Approval Process at Naval Medical Center San Diego. Mil. Med..

[151] Vilasdechanon S., Sopadang A. (2018). Business Process Reengineering for the Saline Management in Hospitals. 2018 5th International Conference on Industrial Engineering and Applications (ICIEA), Singapore, 26–28 April 2018.

[152] Ahmed M., Jones E., Redmond E., Hewedi M., Wingert A., Gad El Rab M. (2015). Food production and service in UK hospitals. Int. J. Health Care Qual. Assur..

[153] Marin-Garcia J.A., Garcia-Sabater J.P., Ruiz A., Maheut J., Garcia-Sabater J.J. (2020). Operations Management at the Service of Health Care Management: Example of a Proposal for Action Research to Plan and Schedule Health Resources in Scenarios Derived from the COVID-19 Outbreak. J. Ind. Eng. Manag..

[154] Nicolay C.R., Purkayastha S., Greenhalgh A., Benn J., Chaturvedi S., Phillips N., Darzi A. (2012). Systematic review of the application of quality improvement methodologies from the manufacturing industry to surgical healthcare. Br. J. Surg..

[155] Sharma G.V.S.S., Prasad C.L.V.R.S., Srinivasa Rao M. (2020). Industrial engineering into healthcare—A comprehensive review. Int. J. Healthc. Manag..

[156] Akmal A., Greatbanks R., Foote J. (2020). Lean thinking in healthcare—Findings from a systematic literature network and bibliometric analysis. Health Policy.

[157] Zepeda-Lugo C., Tlapa D., Baez-Lopez Y., Limon-Romero J., Ontiveros S., Perez-Sanchez A., Tortorella G. (2020). Assessing the Impact of Lean Healthcare on Inpatient Care: A Systematic Review. Int. J. Environ. Res. Public Health.

[158] Suárez-Barraza M.F., Miguel-Davila J.A. (2020). Kaizen–Kata, a Problem-Solving Approach to Public Service Health Care in Mexico. A Multiple-Case Study. Int. J. Environ. Res. Public Health.

[159] Nagi A., Altarazi S. (2017). Integration of value stream map and strategic layout planning into DMAIC approach to improve carpeting process. J. Ind. Eng. Manag..

[160] Montoya-Reyes M., Gonzalez-Angeles A., Mendoza-Munoz I., Gil-Samaniego-Ramos M., Ling-Lopez J. (2020). Method Engineering to Increase Labor Productivity and Eliminate Downtime. J. Ind. Eng. Manag..

[161] Rodriguez-Garcia M., McLean-Carranza A.A., Carlos Prado-Prado J., Dominguez-Caamano P. (2016). Managing Waiting Times to Predict No-shows and Cancelations at a Children’s Hospital. J. Ind. Eng. Manag..

[162] Assis Ferreira G.S., Silva U.R., Costa A.L., de Dallavalle Padua S.I. (2018). The promotion of BPM and lean in the health sector: Main results. Bus. Process Manag. J..

[163] Tlapa D., Zepeda-Lugo C.A., Tortorella G.L., Baez-Lopez Y.A., Limon-Romero J., Alvarado-Iniesta A., Rodriguez-Borbon I. M. (2020). Effects of Lean Healthcare on Patient Flow: A Systematic Review. Value Health.

[164] Frichi Y., Jawab F., Boutahari S. (2020). Modeling the Impact of Hospital Logistics on Quality of Care and Patient Satisfaction: Results of a Survey in Three Public Healthcare Facilities in Fez—Morocco. J. Ind. Eng. Manag..

[165] Abdelfattah F.A., Rahman M.S., Osman M. (2015). Assessing the antecedents of customer loyalty on healthcare insurance products: Service quality; perceived value embedded model. J. Ind. Eng. Manag..

[166] Mahmoud A.S., Ahmad M.H., Yatim Y.M., Dodo Y.A. (2020). Key Performance Indicators (KPIs) to Promote Building Developers Safety Performance in the Construction Industry. J. Ind. Eng. Manag..

[167] Crema M., Verbano C. (2016). Safety improvements from health lean management implementation Evidences from three cases. Int. J. Qual. Reliab. Manag..

[168] Singh P. (2019). Lean in healthcare organization: An opportunity for environmental sustainability. Benchmarking Int. J..

[169] Hallam C.R.A., Contreras C. (2018). Lean healthcare: Scale, scope and sustainability. Int. J. Health Care Qual. Assur..

[170] Kovacevic M., Jovicic M., Djapan M., Zivanovic-Macuzic I. (2016). Lean thinking in healthcare: Review of implementation results. Int. J. Qual. Res..

[171] Macedo Gomes A., Senna P., Monteiro A., Pinha D. (2016). Study on techniques and tools used in lean healthcare implementation: A literature review. Braz. J. Oper. Prod. Manag..

[172] Swarnakar V., Singh A.R., Tiwari A.K. (2020). Evaluating the effect of critical failure factors associated with sustainable Lean Six Sigma framework implementation in healthcare organization. Int. J. Qual. Reliab. Manag..

[173] Al Hammadi F., Hussain M. (2019). Sustainable organizational performance: A study of health-care organizations in the United Arab Emirates. Int. J. Organ. Anal..

[174] Jones D., Womack J., Brunt D., Lovejoy M., Shook J. (2011). Seeing the Whole Value Stream.

